# The rise and fall of tobacco smoking and associated rise and fall of coronary atherosclerosis the lethal role of tobacco

**DOI:** 10.3389/fcvm.2023.1267205

**Published:** 2023-10-03

**Authors:** James S. Lawson

**Affiliations:** School of Biotechnology and Biomolecular Sciences, University of New South Wales, Sydney, NSW, Australia

**Keywords:** rise and fall, coronary atherosclerosis, coronary heart disease, tobacco smoking, infections, diets

## Abstract

In this review two new hypotheses are explored, one, that the decline in coronary heart disease is mainly due to a dramatic decline in the prevalence of underlying atherosclerosis and two, that tobacco smoking has been a much greater influence on atherosclerosis than previously estimated. The major outcome of coronary atherosclerosis is myocardial infarction. Between 1900 and 1960 the prevalence of coronary atherosclerosis dramatically rose in young male soldiers. Between 1960 and 2010 the prevalence of coronary atherosclerosis in young US soldiers equally dramatically fell. Understanding the reasons for this rise and fall offers important insights into the causes of atherosclerosis. In 1960 over 50% of US military personnel were tobacco smokers but by 1988 the rate had reduced to 30%. The increased prevalence of coronary atherosclerosis in young soldiers between 1900 and 1960 was mainly due to increased tobacco smoking. An additional influence was an increase in food and sugar consumption. The fall in atherosclerosis between 1960 and 2010 was probably due to a reduction in tobacco smoking and to a lesser extent, control of hypertension and lowering of high serum total cholesterol. In Western populations up to two thirds of the fall in deaths due to myocardial infarction has been shown to be due to declines in the incidence of heart attacks. Based on the data included in this review it is arguable that the main reason for the fall in the incidence of heart attacks is the fall in the prevalence of underlying coronary atherosclerosis. The adverse influences of tobacco have been well documented. However the enormity of these adverse influences has not been recognised. Over 50% of men continue to smoke tobacco in China, Indonesia, Russia and middle eastern countries. Based on the experience of Western countries over half of these men will die of smoking related conditions.

## Introduction

In this communication we explore the potential causes of the rise and fall of coronary artery atherosclerosis by examining trends in risk factors over time. The major complication of atherosclerosis is myocardial infarction. This investigative review is concerned with coronary atherosclerosis not myocardial infarction. In this review priority is given to data from the United States because of the availability of autopsy studies to document coronary artery atherosclerosis that were conducted on young US soldiers killed in wars in Korea (1955), Vietnam (1971) and Iraq and Afghanistan (2012).

In Western societies, atherosclerosis can commence in childhood and be well established in early teenage years (1). Over 50% of US teenagers who were killed between 1987 and 1994 because of accidental trauma or homicide, had atheromatous lesions in their coronary arteries (2). Classical risk factors which include tobacco use, obesity, hypertension, diabetes, high non-HDL cholesterol and elevated waist to hip ratios are invariably absent in these infants, children and teenagers. An exception in children is excess nutrition and in young men excess nutrition plus tobacco smoking.

### The prevalence of coronary atherosclerosis in young male soldiers

**Figure 1:**
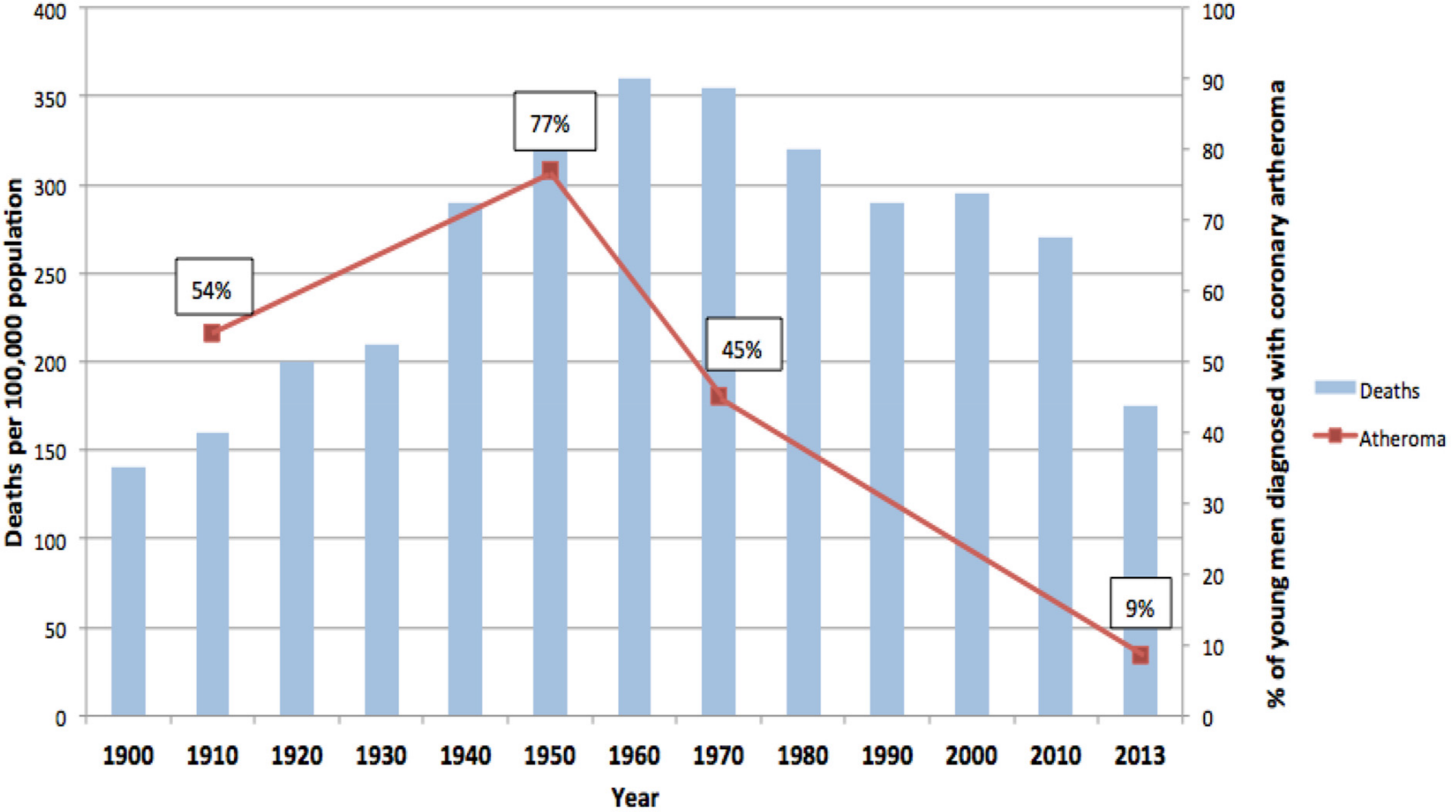
Prevalence of coronary atheroma in young German and American soldiers. Deaths due to coronary heart disease USA. 100 year trends (3–6). Figure reproduced with approval.

The high prevalence of atherosclerosis in young men was first observed in the coronary arteries of 29 of 65 (45%) of young (mean age 27.7 years) German soldiers who had been killed in World War I (3). In an autopsy study the prevalence of coronary atherosclerosis in young (mean age 22 years) United States soldiers killed in 1950 during the Korean war was 231 of 300 (77%) (4). In 1971 the prevalence of coronary atherosclerosis had fallen to 47 of 105 (45%) among young (mean age 22 years) US soldiers in the Vietnam war (5). In 2013 the prevalence had fallen to 345 of 3832 (8.5%) among US soldiers (mean age 26 years) killed in the Iraq and Afghanistan wars (6). More recent information is available from the Iraq and Afghanistan wars where the prevalence of coronary atherosclerosis was 12%–19% based on autopsies of US soldiers of mean age 29 years (7). These declining trends mirror the fall in reported deaths in the United States due coronary heart disease (Figure 1).

Risk factors for coronary atherosclerosis are available from the Webber et al. (2012) study of US soldiers killed in the early years of the Iraq and Afghanistan wars (6). The risk factors and prevalence of coronary atherosclerosis increased with the age of the soldiers (age range 18–59 years). Soldiers aged 40 years and over had 7 times the prevalence of atherosclerosis compared to those aged below 24 years.

### Coronary atherosclerosis in aviation pilots

A similar decline in coronary atherosclerosis has also been observed in both military and civilian pilots killed in aviation accidents (8, 9). The prevalence of coronary atherosclerosis in 42 deceased military pilots aged 20–34 years was 83% pre—1960, and had fallen to 39% of 33 deceased pilots in 1970–1974 (8). The prevalence of minimal to severe coronary atherosclerosis from autopsies for 1996–1999 for general aviation pilots below the age of 30 years was 3 to 4% (9).

It can be argued that these data may not be accurate because the autopsies and assessment of coronary and aortic atherosclerosis were conducted by different pathologists during different times. However atherosclerosis is obvious to a trained pathologist and there is high reproducibility of atherosclerotic grading over decades of time (10, 11). In addition the number of autopsies conducted on these young soldiers is high. We regard the data as being reliable. The diagnostic criteria for coronary heart disease have varied over the years. Despite these problems the trends are obvious (12).

## Myocardial infarction event rates

From 1980 to 2017 age adjusted mortality due to myocardial infarction in global populations fell by 9.7% (13). This fall in mortality could be the result of either a reduction in the fatality associated with myocardial infarction or a reduction in the overall number of myocardial infarction events. In a data based study by Camacho et al. (2022) up to two thirds of the fall in deaths due to myocardial infarction has been shown to be due to falls in event rates, that is, falls in the incidence of heart attacks (14). In the study by Camacho et al. (2022) involving 80.4 million adults in the United Kingdom, Australia, New Zealand and Canada during the 13 years 2002–2015, the incidence of myocardial infarction events in both men and women combined declined by 60% in the UK and Australia, 68% in New Zealand and 69% in Canada (14). In a US based study conducted in 2011 the decline in deaths due to coronary heart disease plateaued and has remained stable (15). The fall in the incidence of heart attacks parallels the fall in coronary atherosclerosis observed in the young US soldiers and the US coronary heart disease mortality rates as shown in Figure 1. Camacho et al. (2022) attributed the decline in myocardial infarction (heart attack) events to the decline in tobacco smoking, reduction in the prevalence of hypertension and the lowering of high serum lipids (14). They did not quantify these specific risk factors.

### Risk factors that may be associated with the rise and fall of coronary atherosclerosis

The evidence suggests that the rise in coronary atherosclerosis in Western (German and US) soldiers from 1915 to 1960 was mainly due to increased tobacco smoking, an improvement in general diets plus an increased consumption of processed sugars. This rise was not due to changes in the prevalence of infectious diseases which declined throughout this time. The fall was probably associated with a substantial fall in smoking tobacco. However there is a need for caution as the evidence based on young soldiers may not hold true for general populations. In addition there have been recent adverse cardiovascular disease trends in the United States (15).

### Tobacco smoking

**Figure 2:**
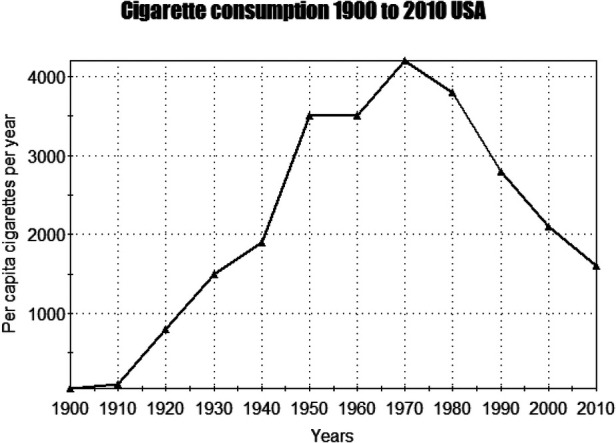
Cigarette consumption 1900 to 2010 USA (16).

As shown in Figure 2 tobacco smoking in the US grew rapidly from 1900 until a peak prevalence in 1964 (16). During the first world war (1914–1918) the smoking of cigarettes among both the military and general populations dramatically increased. US General John J. Pershing encouraged smoking among soldiers when he stated “You ask me what we need to win the war? I answer, tobacco as much as bullets” (17). For US military veterans smoking had lethal consequences. Rogot & Murray (1980) in a 16 year follow up of these veterans, demonstrated that smokers were at 1.7 times greater than expected risk of death (18). The leading causes of smoking related deaths were emphysema (14.8 times greater than expected), lung cancer (11 times), aortic aneurism (5 times) and coronary heart disease (1.6 times) (18).

It is probable that over 50% of United States military personnel were regular tobacco smokers during and after the second world war (1942–1960). This was confirmed by surveys conducted in 1980 which showed that 51% of the US military personnel smoked cigarettes (19). However, by 1998 smoking rates among military personnel had reduced to 30% (19). These smoking patterns among the military were broadly similar to those among US civilian males among whom the prevalence of smoking fell from 57% in 195 to 51% in 1966 (20).

Based on twelve prospective studies the risk of sudden death due to coronary heart disease is 3 fold greater among smokers as compared to non-smokers and past smokers (21). Probably the most famous of these studies is that of Doll et al. (1994) who in a 40 year follow up study of 34,439 medical doctors in the United Kingdom demonstrated that about 50% of regular tobacco smokers would be killed by their habit (22). In a United Kingdom study of 14,000 cases of non- fatal myocardial infarction and 32,000 controls 80% of myocardial infarctions at ages 30–49, 66% at ages 50%–59% and 50% at ages 60–79 were caused by tobacco smoking (23).

Tobacco smoking has a harmful effect on vascular endothelial cells which leads to early atherosclerosis (24, 25). Smoking tobacco also leads to platelet adherence which provokes the development of coagulation and inflammation (24).

The prevalence of tobacco smoking has declined in most Western countries. However the incidence remains high in many countries with a smoking prevalence among males above 50% in China, Indonesia, Jordan, Turkey, Russia and Greece (26).

Tobacco smoke is an independent risk factor for atherosclerosis. The mechanisms of tobacco smoke and atherosclerosis have been recently reviewed in detail by Klein and Fu et al. (27, 28). Smoking may initiate and accelerate atherosclerosis by damaging vascular intima endothelial cells leading to oxidative stress, thrombosis, lipid abnormalities and inflammation. Nicotine in tobacco smoke causes dysfunction of endothelial and vascular smooth muscle cells, oxidative stress and abnormal lipid metabolism.

### Hypertension

Hypertension has long been known to increase the extent and severity of atherosclerosis in both humans and experimental animals (29). Hypertension prevalence trends prior to 1950 are not available. However death rates from strokes in the US consistently declined from 1900 to 1940 (30). As strokes are associated with hypertension it can be assumed that the prevalence of hypertension did not increase during this period. In the US the age adjusted prevalence of hypertension (blood pressure equal or over 140 mmHg systolic, 90 mmHg diastolic) declined from 29.7% in 1960 to 20.4% in 1991. This favourable trend was reversed from 25% in 1994 to 28.6% in 2002.

The decline in prevalence of hypertension between 1960 and 1991 may have had a modest influence on the decline in the prevalence of coronary atherosclerosis.

### Cholesterol

High plasma levels of low density lipoprotein cholesterol and low plasma levels of high density lipoprotein cholesterol are associated with atherosclerosis (31).

Serum cholesterol levels between 1900 and 1950 are not available because the data was not systematically collected until after the pioneering studies of Ancel Keys in 1957 (32). However the food consumption patterns in the US remained largely unchanged during this period (33). As serum cholesterol levels are largely dependent on patterns of food consumption it is reasonable to assume there were no major changes in serum cholesterol levels from 1900 to 1950 (32).

Between 1976 and 2006 total serum cholesterol levels in the US fell from 210 to 200 mg/dl (34). Body mass index increased from 26 to 29 kg/m^2^ during this latter period and the prevalence of obesity doubled (34).

The early (1976–2006) falls in total cholesterol have been largely attributed to changes in diet (35). Later more modest changes have been attributed to the widespread use of statins in the US where 25% of adults aged 40 years and over take statins (36). 24% of the fall in US coronary heart disease mortality rates between 1980 and 2000 have been attributed to falls in total serum cholesterol (37).

### Food

Food consumption patterns in the US between 1900 and 1950 are relevant because of the rise of vascular atherosclerosis and coronary heart disease deaths during these decades. The details of these food consumption patterns have recently become available (Table 1) (31). With three exceptions, during the 50 year period from 1900 to 1950, the composition of diets of Americans remained mostly unchanged (33). The three exceptions were (i) a marked rise in consumption of refined cane sugar, (ii) a decline in consumption of fresh fruit (no data from 1900 is available for fresh vegetables) and (iii) an increased consumption of eggs. However there was an apparent overall increase in the average food consumption of Americans as judged by the an average increase in both heights and weights as shown in Table 2.

**Table 1:** United States dietary trends—1900–2000 (33).

Foods—annual per capital	1900	1950	2000
Total Kcalories	3,200	3,200	4,100
Saturated fats grams	50	50	55
Total fats grams	120	140	190
Red meat pounds	154	136	120
Sweeteners—1900–1950 mainly cane sugar. 1950–2000 cane plus corn sugars pounds	65	110	149
Poultry pounds	22	24	77
Dairy products pounds	458	411	382
Fresh fruit pounds	220	104	129
Fresh vegetables pounds	na	123	186
Eggs pounds	33	49	32

**Table 2:** Fifty year trends in heights and weights of United States males (45, 46).

Average heights	1917/1918	1946
US soldiers	67.5 inches	68.4 inches
US college students	68 inches	70 inches
US military males	67 inches (1896)	70 inches (1996)
Average weights	1885–1900	1955
US males 68 inches tall	154 pounds	161 pounds

In the US during the period 1900–1950, there was little change in the patterns of consumption of saturated and other fats (33). Accordingly it is unlikely these fats influenced the increase in atherosclerosis and heart disease during this period.

Bechtold et al. have conducted a recent review of the intake of specific food groups and the risk of coronary heart disease (38). The data is based on meta-analyses of each food group. The most harmful food group includes drinks with refined sugar. Processed meats may also be harmful but the confidence interval is wide precluding definite conclusions. This confirms the original observation in the Seven Countries studies by Keys et al. that sweet sugar products were associated with coronary heart disease (39). Prospective studies among US men and women indicate that high intakes of sugar sweetened beverages increase the risk of coronary heart disease by 20% (40, 41). US women who consume sugar sweetened beverages have an up to 35% increased risk of coronary heart disease (41). US men who consume sugar sweetened beverages have an up to 20% increased risk of coronary heart disease (40). These findings are based on long term (24 and 22 years respectively) prospective studies (40, 41). This evidence is of moderate quality.

### Heights and weights

Height and weights can be an indication of food consumption particularly during infancy, childhood and young adult hood (42). As shown in Table 2 both heights and weights of US males gradually increased during the 50 year period from about 1900 until 1955. In 1885/1900 4% of US males aged 20–29 years were at least 6 feet tall compared to 1955 when 20% of comparable males were at least 6 feet tall (40). This suggests that there had been an average increase in food consumption in the United States during these decades. The increase in heights and weights would also be influenced by the fall in childhood infections during this same period (43).

There is a significant association between body weight and cardiovascular disease including coronary heart disease. This is demonstrated by the 40 year follow up of 2.3 million adolescent Israelis among whom overweight and obesity were strongly associated with an increased hazard ratio of up to 4.9 for cardiovascular mortality in adulthood (44).

Based on this evidence it can be assumed that changes to food consumption patterns in the US from 1900 to 1950 could be associated with an increased prevalence of atherosclerosis. It is unlikely that food consumption patterns are associated with the fall in coronary atherosclerosis from 1950 until 2000 because total calories, total fats and refined sugar consumption all increased (Table 1). This evidence is of middle ranking quality as the evidence is based on associations not direct evidence. The influence of these changing food consumption patterns on atherosclerosis cannot be quantified.

## Infection

**Figure 3:**
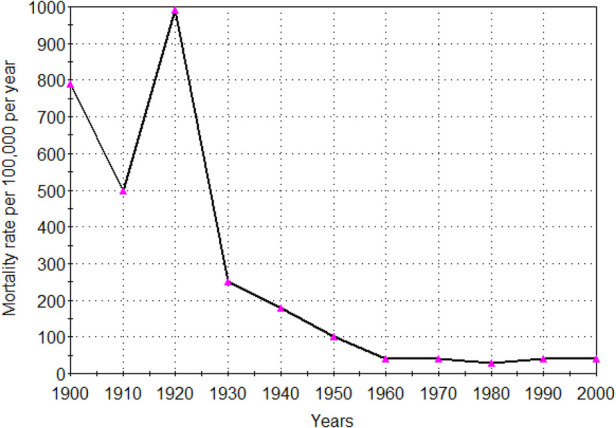
Infectious disease mortality rate adults (18 years and over) United States 1900–2000. Age adjusted (48, 49).

While infections may have a role in the initiation of atherosclerosis in child hood and during later life they do not appear to have influenced the rise and fall of coronary atherosclerosis (47). There was a continuous decline in deaths at all ages due to infectious diseases during the 50 years 1900–1950 the same period as the continuous rise in atherosclerosis and coronary heart disease (48). Deaths due to infectious disease continued to decline during the next 50 years 1950–2000. Coronary atherosclerosis and coronary heart disease also declined during this latter period. Adult mortality rates due to infectious diseases from 1900 to 2000 are shown in Figure 3.

Prior to the introduction of vaccines, diphtheria, pertussis, polio, measles, mumps, rubella and hepatitis A were common infections in the US. Measles was almost universal among children. These infections were virtually eliminated between 1948 and 2005 following the introduction of vaccines (48). Measles, the most common of the relevant infections, was virtually eliminated by 1970. Respiratory and gastro- intestinal infections are also very common among children but have not been eliminated to the same extent as the vaccine sensitive infections.

The decline in all age deaths due to infections occurred in the virtual absence of vaccines and antibiotics before 1950 (49). This decline in deaths due to infection before 1950 has been attributed to an increase in education, living standards and safe water supplies (43). The increased prevalence of atherosclerosis in young Western subjects during this same time would not have been due to a fall in childhood or adult infectious diseases.

The elimination of common childhood infections which occurred between 1948 and 1995, parallels the reduction in coronary atherosclerosis (50). However in a Japanese prospective study of subjects, none of whom had vaccinations, both measles and mumps infections were significantly associated with an approximate 20% reduced risk of atherosclerotic cardiovascular disease (51). This Japanese study confirmed the findings of a previous Finnish study which also showed that childhood contagious infections such as measles, mumps and rubella were associated with an approximate 20% reduction in risk of coronary heart disease (52). In this same study there was a significantly increased risk of coronary heart disease associated with childhood enterovirus, herpes simplex virus and Chlamydia pneumoniae infections (52). Accordingly it is reasonable to assume that the introduction of measles, mumps and rubella vaccines during the 1960s were not responsible for lowering the prevalence of atherosclerosis. This evidence is suggestive not conclusive.

### Infections combined with food and atherosclerosis

There is epidemiological based evidence which indicates that infections alone do not lead to atherogenesis. This is based on the experience of “native” populations who have a high burden of infections and negligible atherosclerosis and coronary heart disease. Current studies of “native” populations using modern methods have been conducted among Amazon river communities by Kaplan et al. and in Ghana by Koopman et al. (53, 54). There is an increase in cardiovascular disease when “native” populations begin to consume Western diets (54). This evidence is consistent.

In a recent prospective study with a 4 year follow up of 63,411 Korean women by Joo et al. (2021) there was a significant association between human papilloma virus (HPV) infections and the onset of cardiovascular disease with a hazard ratio of 1.69 (CI 1.19–2.51) (55). The prevalence of cardiovascular (CVD) increased in parallel with increased weight. The increased weight could be considered as an indication of increased food consumption. While this evidence is consistent with previous studies which indicate an association between food consumption patterns, infections and atherosclerosis, the evidence is suggestive not conclusive.

## Discussion

Between 1900 and 1960 there was an unprecedented rise in the prevalence of coronary atherosclerosis and a parallel rise in deaths due to coronary heart disease. Between 1960 and 2020 there was an equally unprecedented fall in both coronary atherosclerosis and coronary heart disease mortality. The evidence indicates that it is highly likely the prevalence of tobacco smoking is largely responsible for both the rise and fall in the prevalence of coronary atherosclerosis. In addition to the decline in coronary atherosclerosis, statins, antithrombotics, antihypertension agents and surgical interventions have been responsible for the decline in coronary heart disease mortality (14).

The large increase in tobacco smoking during the period 1900 to 1960 is well documented (US Surgeon General. Centers for Disease Control and Prevention 2014). The initiation and exacerbation of both coronary atherosclerosis and coronary heart disease by tobacco smoking is extensively documented (56, 57).

Camacho et al. have shown that up to two thirds of the fall in deaths due to coronary heart disease were due to falls in event rates (that is heart attacks) (14). This conclusion is based on 1.95 million events in 80.4 million people in four countries. These countries were the United Kingdom, Canada, New Zealand and Australia. In simple terms, in recent decades many fewer people were having heart attacks. Based on the data included in this review it is arguable that this is because of the fall in prevalence of underlying coronary atherosclerosis which in turn is due to the reductions in tobacco smoking in the populations studied by Camacho et al. (14).

Despite the substantial fall in atherosclerosis and coronary heart disease mortality, these problems remain as major public health issues.

### Limitations of the evidence

In this investigative review the ecological fallacy must be considered. Ecological fallacy arises from thinking that relationships observed for groups necessarily hold for individuals. The most famous is the fallacy committed by Emile Durkheim in 1897 who concluded that Protestants in Prussia committed suicide at 8 times the rates of Catholics (58). He failed to recognise that the groups he studied were mixtures of Protestants and Catholics. The real difference was two fold.

In this current review there are a number of limitations which could lead to fallacious conclusions. (i) young male soldiers are not representative of the general population, (ii) male behaviour differs from females, (iii) soldiers tend to smoke and drink alcohol more than civilian counterparts (iv) food consumption patterns and infections based on populations may differ when based on individuals. While the prevalence of coronary atherosclerosis in young soldiers was based on individual autopsies there may be differences in the assessments by different pathologists over many decades.

### Strengths

Despite these limitations the 100 year trends in the prevalence of atherosclerosis, coronary heart disease and infectious diseases are all well established. These trends are based on studies of individuals and do not have the risk of ecological fallacies.

## Conclusions

In this review two new hypotheses were explored, one, that the decline in coronary heart disease has been mainly due to a dramatic decline in underlying atherosclerosis and two, that tobacco smoking has been a much greater influence on atherosclerosis than previously estimated.

Although the evidence is based on associations it is highly likely that the dramatic rise in coronary atherosclerosis and accompanying coronary heart disease in Western communities was due to the influences of tobacco smoking. It is equally likely that the fall in atherosclerosis has been mainly due to the fall in tobacco smoking.

Increased consumption of refined sugars and improved diets are likely to have also contributed to the rise in atherosclerosis between 1900 and 1950. However sugars and food are not likely to have contributed to the fall in atherosclerosis as there has been an increase in their consumption in Western communities from 1950 to 2000.

When the observations in this review are considered together with the experimental and epidemiological evidence, tobacco is confirmed as a lethal substance. The adverse influences of tobacco have long been documented. However the enormity of these adverse influences is still not completely recognised. Over 50% of men continue to smoke tobacco in China, Indonesia, Russia and middle eastern countries (59). Based on the experience of Western countries over half of these men will die of smoking related conditions.

It should be emphasised that Table 1 contains US data. Food consumption patterns and their associations with coronary heart disease were substantially different in other countries (39).
